# Significant Changes in Bacterial Communities Associated with *Pocillopora* Corals Ingestion by Crown-of-Thorns Starfish: An Important Factor Affecting the Coral’s Health

**DOI:** 10.3390/microorganisms10020207

**Published:** 2022-01-19

**Authors:** Zhenjun Qin, Kefu Yu, Shuchang Chen, Biao Chen, Qiucui Yao, Xiaopeng Yu, Nengbin Pan, Xuelu Wei

**Affiliations:** 1 Guangxi Laboratory on the Study of Coral Reefs in the South China Sea, Coral Reef Research Center of China, School of Marine Sciences, Guangxi University, Nanning 530004, China; qzj_gxu@163.com (Z.Q.); 2027301002@st.gxu.edu.cn (S.C.); biaochenwork@163.com (B.C.); yaoqiufeng504@163.com (Q.Y.); xiaopengyu@st.gxu.edu.cn (X.Y.); 2127301027@st.gxu.edu.cn (N.P.); 2127301033@st.gxu.edu.cn (X.W.); 2 Southern Marine and Engineering Guangdong Laboratory (Zhuhai), Zhuhai 519080, China

**Keywords:** bacterial community, Symbiodiniaceae density, *Pocillopora*, crown-of-thorns starfish, corallivores

## Abstract

Coral ingestion by crown-of-thorns starfish (COTS) is an important cause of coral reef degradation, although the impacts of COTS feeding on coral-associated microbial communities are not well understood. Therefore, in this study, we analyzed the coral tissue-weight, Symbiodiniaceae density (SD), bacterial community composition, and the predicted functions of bacterial genes associated with *Pocillopora* corals in healthy portions and feeding scars, following COTS feeding. Coral tissue-weight loss rate in the feeding scars was 71.3–94.95%. The SDs were significantly lower in the feeding scars, and the SD-loss rate was 92.05% ± 2.12%. The relative abundances of bacterial communities associated with *Pocillopora* corals after COTS feeding changed significantly and were almost completely reorganized at the phylum and genus levels. Analysis of the microbial metagenomic-functional capacities showed that numerous physiological functions of the coral-bacterial holobionts in the feeding scars were different, including amino acid metabolism, xenobiotic biodegradation and metabolism, lipid metabolism, membrane transport, signal transduction, and cell motility, and all these capacities could be corroborated based on metagenomic, transcriptomic or proteomic technologies. Overall, our research suggests that coral holobionts may be destroyed by COTS, and our findings imply that bacterial communities in feeding scars could affect the health of *Pocillopora* corals.

## 1. Introduction

Since the 1980s, most coral reefs worldwide have been threatened and have undergone rapid degradation [1,2]. Many factors cause coral reef degradation, mainly including human destructive activities (e.g., overfishing and pollutant discharge), climate change (e.g., abnormally high temperatures), the emergence of coral diseases, and damage from coral predators [2,3,4,5]. Among these factors, damage from coral predators is a particularly important factor in coral reef degradation. It is well known that many aquatic organisms are coral predators, including crown-of-thorns starfish (COTS, *Acanthaster planci*), sea urchins, *Drupella* spp., members of the family Labridae, etc. [6,7,8]. Among these predators, crown-of-thorns starfish (COTS) is a greatly harmful predator to coral reefs, and damages corals far more than other coral predators [9]. For example, four COTS outbreaks occurred in 1962, 1979, 1993, and 2010 in the Great Barrier Reef (GBR) [10], and damage from COTS has been extensive and frequent in many areas around the world [11,12,13]. The average live coral cover (LCC) of the GBR dropped from 28.0% in 1982 to 13.8% in 2012, and 47.5% of the live coral loss was directly caused by COTS outbreaks [10]. Similarly, COTS outbreaks have also occurred in Guam [13], southern Japan [11], and French Polynesia [11] in recent decades, with a large number of corals lost and rapid decreases in the LCC. For example, corals dominate a healthy reef with a LCC > 40% in the Moorean outer-reef, but algae colonize dead coral skeletons following severe predation by COTS (~10% LCC) [11]. In this case, mostly dead and weakened coral skeletons were swept away by a cyclone occurring at the end of the COTS outbreak and colonizing algae once again dominate the devastated reef (~5% LCC) [11].

COTS is a carnivorous animal whose main food is reef-building corals. It can feed on a large area of coral surface tissue in a short time. Each COTS can consume hundreds of square centimeters of coral tissue per day [14]. When feeding on corals, COTS wraps its extrudable stomach around the corals [15], after which gastric secretions digest the coral tissues within 3–5 h and large feeding scars are left on the corals [10,16,17]. For some encrusting corals (such as *Montipora* corals) and massive corals (such as *Favia* and *Favites* corals), COTS wraps the entire coral surface, thereby feeding on the entire coral and causing it to die [15]. In contrast, for some branching corals, COTS can only cover the outside coral branches, and the inner branches often survive, resulting in partial feeding scars [15]. At present, the direct impact of COTS on corals is generally assessed by performing macro-ecological surveys, that is, the area and numbers of feeding scars are counted through underwater video and photographs [12,18,19], but the effects on coral-associated microorganisms after COTS feeding remain unknown.

Scleractinian corals rely on the symbiotic relationship between coral hosts and microorganisms, which enables corals to survive and thrive in the oligotrophic tropical ocean [20,21]. Coral holobionts contain a variety of microorganisms, including bacteria, archaea, fungi, viruses, and Symbiodiniaceae [22]. These microorganisms live in coral mucus, tissues, and skeleton, and cooperate with the host to support its physiological functions (including biogeochemical cycling, material transformation, antibacterial defenses, and nutrient acquisition), thereby helping to maintain the health of the coral reef ecosystem [21,23,24]. Among these microorganisms, Symbiodiniaceae play an important role in coral holobionts, providing ~95% of the energy of coral hosts [1,3]. In addition, the associated bacteria are highly diverse and complex microorganisms [22,25]. Some coral-associated bacteria, such as *Endozoicomonas*, are considered vital to coral health and play important roles in the responses of holobionts to environmental stresses [25,26]. The high abundance and diversification of coral-associated bacterial communities can help maintain the microbial flexibility of the coral holobionts to adapt to the changing environment [27,28]. In the case of coral-bacterial symbionts, some bacteria are beneficial to the coral host [22,29,30]. For example, the photosensitive-associated microorganisms Oceanospirillales and Halomonas, which promote dimethyl sulfoniopropionate metabolism and yielding products used in the metabolism of the host, may enable the coral holobionts to more efficiently carry out energy recycling [22,31]. *Endozoicomonas* is a good indicator candidate of the health state of corals; massive losses of these bacteria would have a serious negative impact on the physiological functions of the holobionts [32,33]. In contrast, certain bacteria are potential pathogens, such as some species in the *Vibrio* genus (e.g., *Vibrio shiloi* and *Vibrio coralliilyticus*). An increased abundance of these bacteria may lead to poor health of corals [34,35,36]. Therefore, exploring changes in coral-associated bacterial communities can provide a basis for assessing the health state of corals. However, the effect of COTS ingestion on the coral-associated bacterial communities, physiological functions, and health states of coral holobionts are currently unknown.

The *Pocillopora* genus is a widespread genus in the coral reef regions of the Indo-Pacific and commonly found in the tropical coral reefs of the South China Sea (SCS). Adult *Pocillopora* corals are usually >20 cm wide and these colonies are covered with different types of verrucae. Colony branches are thick and compact in habitats exposed to strong waves, whereas they can become open and thinner in protected habitats [37]. Colonies occur in most shallow water environments, ranging from exposed reef fronts to protected fringing reefs [37]. The Xisha Islands (also known as the Paracel Islands, 15°40′ N–17°10′ N, 110° E–113° E) are located in the central SCS. More than 20 tropical atolls and islands of various sizes are distributed in the SCS, and they have a high diversity of reef corals and numerous scleractinian coral species [38,39]. Previous macro-ecological surveys showed that *Pocillopora* is one of the most dominant coral genera of the Xisha Islands [38]. By conducting an in-situ investigation of the Passu Keah atoll of the Xisha Islands in 2019, we found that many COTS fed upon *Pocillopora* corals and that the outer-side branches were ingested, whereas the inner branches were in a healthy state. By comparing the associated microorganisms in the feeding scars and the healthy portions of the *Pocillopora* corals, it is possible to better understand the damage to coral holobionts caused by COTS feeding, as well as the healthy state.

In this study, samples of *Pocillopora* corals with COTS feeding scars and healthy portions were collected from the outer reef slopes of Passu Keah, Xisha Islands. The coral tissue-weight loss rate, Symbiodiniaceae density (SD) associated bacterial compositions, microbial-metagenomic functional capacities of healthy corals, and coral feeding scars were analyzed to explore the damage and impact of COTS feeding on coral holobionts.

## 2. Materials and Methods

### 2.1. Study Site and Coral Collection

Research sites were located in Passu Keah, Xisha Islands (16°01′19′′ N–16°04′25′′, 111°44′30′′ E–111°49′47′′ E). Passu Keah is ~330 km from Hainan Island and is an atoll with ~26 km^2^ of reef area. During our in-situ coral reef ecological survey conducted in 2019, we found that many COTS were feeding upon corals, including *Pocillopora* corals. COTS were ingesting the top portions of many *Pocillopora* corals, whereas less ingestion occurred in the groove portions. *Pocillopora* coral fragments were divided, separating feeding scars and healthy portions, by hammer and chisel from a depth range of ~6 m via SCUBA diving and immediately collected with sample bags. A total of 38 *Pocillopora* coral specimens (25–30 cm^2^ per sample) was collected in this study (i.e., 19 coral colonies were sampled, and each of these collected samples originated from the same coral colony). The distance between coral colonies was above 10 m. Feeding scar samples included skeleton and a bit of tissue and mucus left after COTS ingestion. All coral samples were cleaned in the boat with artificial sterile seawater (35‰ salinity) to ensure that they were not contaminated with free-living bacteria or Symbiodiniaceae. Each specimen was then divided into two portions, where one portion (~20 cm^2^) was used for SD determination and the other portion (~5 cm^2^) was used for bacterial DNA extraction. All the total *Pocillopora* coral specimens (i.e., 38 specimens used for SD determination and 38 specimens used for bacterial DNA extraction) were preserved in liquid nitrogen and immediately transported to our laboratory.

### 2.2. Coral Tissue-Weight Loss Rate and Symbiodiniaceae Density Determination

Coral samples were weighed using an electronic balance (BSM120.4, accuracy 0.0001 g, Shanghai Zhuojing Electronic Technology Co., Ltd., Shanghai, China) before coral tissue was removed. A Waterpik^TM^ (3–5 kgf cm^−2^) was then used to remove coral tissue using artificial sterile seawater (35‰ salinity) until only white coral skeleton remained (1–3 min). At this point, the organization of the coral tissue was considered to have been completely removed. Coral skeletons were dried in a drying cabinet at 80 ℃ for 48 h and reweighed using the electronic balance. The volume of the initial coral tissue and Symbiodiniaceae slurry of feeding scars and healthy portions was measured in a graduated cylinder; subsequently, the sample was homogenized and subsampled into four 3-mL aliquots, and centrifuged at 4000 rpm for 5 min. A total of 38 small coral fragments were collected, and Symbiodiniaceae measurements were conducted on feeding scars and healthy portions. SDs of feeding scars and healthy portions were calculated based on replicate hemocytometer counts (*n* = 8), and surface areas of feeding scars and healthy portions were determined based on the correlation between the weight of the aluminum foil used to cover each sample and the surface area [40,41]. The wet weight difference of the coral sample with tissue and the skeleton washed and dried, combined with the surface area, was calculated to determine the coral tissue per unit area as well as the tissue loss ratio in feeding scars.

### 2.3. DNA Extraction, Polymerase Chain Reaction (PCR) Amplification, and Illumina MiSeq Sequencing

Coral fragments (~50 mg), including tissue, mucus, and skeleton, were cut with scissors, and then total genomic DNA was extracted using the FastDNA^®^ SPIN Kit for Soil (MP Biomedicals, Irvine, CA, USA) according to the manufacturer’s recommended procedures. To minimize individual random errors, each coral sample was tested for total genomic DNA by taking six replicate measurements. After quality and purity checks, the extracted DNA samples were used as a template for PCR. The barcode forward primer 338F (5′-ACTCCTACGGGAGGCAGCAG-3′) and the reverse primer 806R (5′-GGACTACHVGGGTWTCTAAT-3′) were used to amplify the V3–V4 region of the bacterial 16S rRNA gene [42]. 20 µL reactions which contains ∼50 ng of DNA and 25 μL of 2X TransStart FastPfu DNA Polymerase were prepared using a TransGen AP221-02 PCR Kit (), and the reactions were run on an ABI GeneAmp^®^ 9700 thermal cycler (Thermo Fisher Scientific Co., Ltd., Waltham, MA, USA). PCR was described by Qin et al. [43]. Purified replicating amplicons of the same coral samples were pooled in equimolar amounts and paired-end sequencing was performed on the Illumina MiSeq platform according to standard protocols (2 × 300, Majorbio Bio-Pharm Technology Co., Ltd., Shanghai, China). Raw reads were deposited into the NCBI Sequence Read Archive database (Accession Number: PRJNA661173).

### 2.4. Data Analysis

PEAR software was used to merge the overlapping paired-end (PE) reads into 16S tags. This allowed for generation of the full-length 16S V3–V4 sequence for coral-associated bacteria 16S read analysis [44]. The raw sequences of coral-associated bacteria obtained from Illumina MiSeq sequencing were analyzed using the Trimmomatic (v0.33) software platform to exclude reads with homopolymer inserts greater than six base pairs (bp) and low-quality tail scores (<20), after setting a quality window of 50 bp [45]. After removing chimeric sequences, operational taxonomic units (OTUs) were clustered with a 97% similarity cut-off using UPARSE v7.1 (https://sourceforge.net/projects/rdp-classifier/, accessed on 20 April 2020). The taxonomy of each 16S rRNA sequence was identified and classified using the Ribosomal Database Project (RDP v2.2) by setting a bootstrap confidence level of 0.7; a representative sequence for each OTU was obtained. The SILVA database (http://www.arb-silva.de, accessed on 20 April 2020) was used for the 16S rRNA OTU alignment-based RDP classifier method using QIIME (version 1.9.1; http://qiime.org/scripts/assign_taxonomy.html, accessed on 20 April 2020) [46]. Similarity percentage (SIMPER) analysis was performed to examine which OTU contributed most to the dissimilarity among coral samples of healthy portions and feeding scars. Based on the clustered OTUs, alpha diversity indices including ACE, Chao 1, and Shannon-Wiener indices were calculated by Mothur (version v.1.30.1). Beta diversity was analyzed by principal co-ordinates analysis (PCoA), permutation multifactorial analysis of variance (PERMANOVA) was carried out at the OTU level to determine significant differences, and the analysis of similarity (ANOSIM) was based on unweighted UniFrac distances. Significant differences in coral-associated bacterial communities were tested by PERMANOVA with 9999 permutation-based Bray-Curtis dissimilarity matrix in R (vegan package; http://www.R-project.org/, accessed 20 April 2020) [47].

The predicted functions of bacterial genes were inferred from 16S rRNA marker gene sequences using the Phylogenetic Investigation of Communities by Reconstruction of Unobserved States (PICRUSt, V1.0.0) computational approach, based on information from the Clusters of Orthologous Groups (COG) of proteins, and the Kyoto Encyclopedia of Gene and Genomes (KEGG) databases (https://picrust.github.io/picrust/, accessed 20 April 2020) [48]. Predicted functional-composition profiles were collapsed into the hierarchical level 2 categories of the KEGG database pathways. The relative abundance of each coral specimen’s functional traits was calculated using Statistical Analysis of Metagenomic Profiles (STAMP v2.1.3) [49].

Levene’s test, Durbin-Watson’s test, and Shapiro-Wilk’s test were used to assess whether the data met the assumptions of homogeneity, normality, and independence, respectively. Student’s *t*-test was used to compare SD and the relative abundances of all level 2 metabolic pathways in the KEGG database between coral samples collected in coral feeding scars and healthy portions. This test was also used to determine whether the alpha diversity and beta diversity in feeding scars and healthy portions were statistically significant. All data are presented as mean ± standard deviation. The statistical significance level was set at *p* < 0.05 for all analyses. All multidimensional statistical analyses were performed in the R software environment (R 3.1.2) using the vegan package (vegan package; http://www.R-project.org/, accessed 20 April 2020). The heat map was also plotted under the R platform.

## 3. Results

### 3.1. Symbiodiniaceae Density and Coral Tissue-Weight Loss Rate

**Figure 1:**
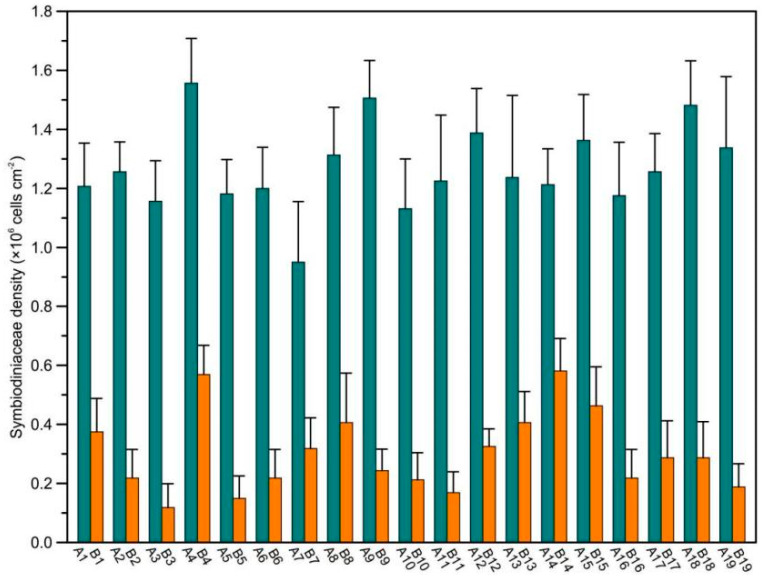
Coral-symbiotic Symbiodiniaceae density (SD) of *Pocillopora* corals between healthy portions and feeding scars ingested by crown-of-thorns starfish in the central South China Sea (SCS).

In this study, 38 *Pocillopora* coral specimens were collected for SD determination in healthy portions and feeding scars. SDs were significantly different between corals in healthy portions (range: 0.95 ± 0.21–1.56 ± 0.15 × 10^6^ cells cm^−2^) and feeding scars (range: 0.12 ± 0.01–0.58 ± 0.11 × 10^6^ cells cm^−2^; Figure 1, Table S1, Student’s *t*-test, *p* < 0.001). After COTS ingestion, the SD-loss rate of *Pocillopora* was 92.05 ± 2.12%. Additionally, the wet weight of coral tissue was significantly different between corals in healthy portions and feeding scars (Table S1, Student’s *t*-test, *p* < 0.001); the coral tissue-weight loss rate in the feeding scars was 71.3–94.95%. In summary, after COTS ingestion, the SDs of *Pocillopora* corals greatly decreased. In-situ observations showed that the white skeletons of *Pocillopora* coral were exposed.

### 3.2. Alpha-Diversities of Coral-Associated Bacterial Communities

After quality filtering, a total of 1,741,939 16S rRNA gene reads were identified (34,103–64,381 reads per sample), and these reads were clustered into different microbial OTUs at a threshold of 97% identity (Table S2). The average length of these sequences was 440 bp. The Good’s coverage of each sample library exceeded 99%, indicating that these sequencing results represented real bacterial community conditions (Good 1953). The average number of bacterial OTUs identified in the feeding scars (1831 ± 338 OTUs) was significantly higher than that in the healthy portions (424 ± 133 OTUs) (Student’s *t*-test, *p* < 0.001).

Diversity was significantly different between the feeding scars and healthy coral portions (Table S2). For example, the mean Shannon-Wiener (H’) index of the feeding scars was 5.28 ± 0.55, whereas it was only 2.04 ± 0.51 in the healthy portions (Student’s *t*-test, *p* < 0.001). Other indices, including the Ace, and Chao 1 indices, were also significantly different between healthy portions and feeding scars. The Ace and Chao 1 indices were significantly higher for the feeding scars than for the healthy portions (Ace: 595.58 ± 182.00 vs. 2443.2 ± 428.63, Student’s *t*-test, *p* < 0.001; Chao 1: 567.93 ± 178.85 vs. 2416 ± 430.2, Student’s *t*-test, *p* < 0.001).

### 3.3. Taxonomic Compositions of Bacteria

**Figure 2:**
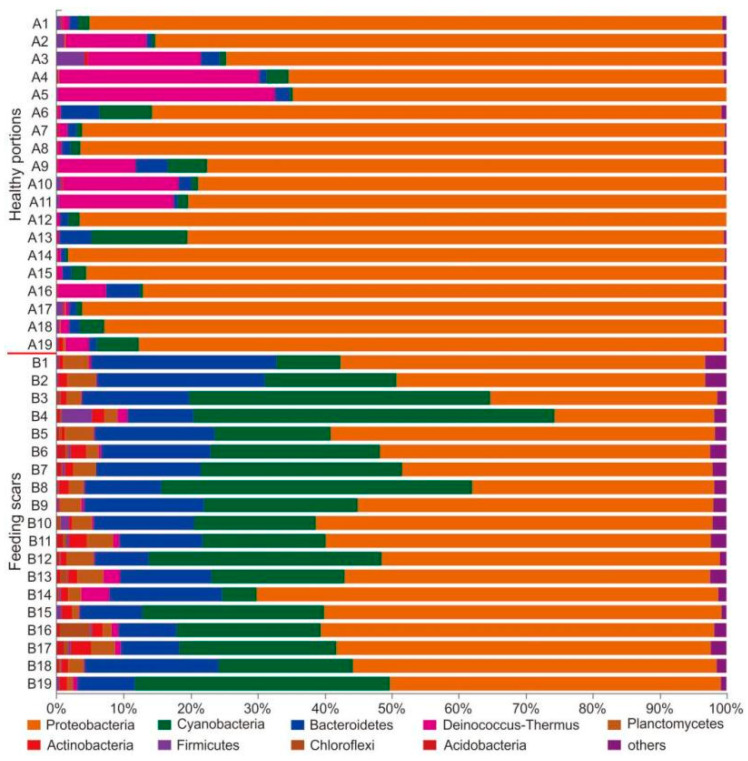
Relative abundances of coral-associated microbial community members in *Pocillopora* sampled from healthy portions and feeding scars at the phylum level, as determined using the RDP classifier.

**Figure 3:**
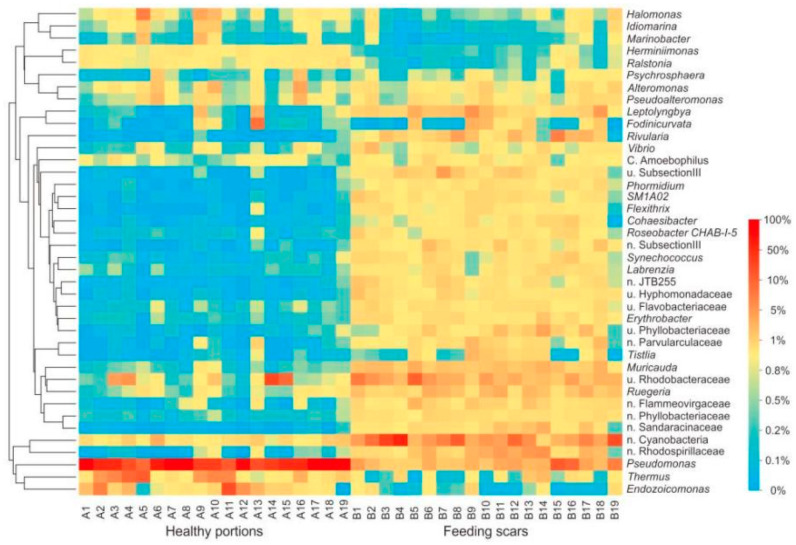
Heatmap showing the percentage of the 40 most abundant microbial genera associated with *Pocillopora* sampled from healthy portions and feeding scars.

The total taxonomic compositions of bacteria identified in the analyzed samples included 46 phyla, 109 classes, 317 orders, 606 families, 1429 genera, and 2779 species. The taxonomic compositions were significantly different between coral specimens collected in the healthy portions and feeding scars (Figure 2 and Figure 3). At the bacterial phylum level, Proteobacteria (85.69% ± 10.50%), *Deinococcus-Thermus* (8.12% ± 10.28%), Cyanobacteria (2.94% ± 3.50%), and Bacteroidetes (2.16% ± 1.65%) were absolutely dominant (average relative abundance >1%) in all samples collected from healthy portions. Although the *Deinococcus-Thermus* and Cyanobacteria exhibited highly variable relative abundances among healthy tissue samples, these bacteria were detected in every coral sample. In contrast, the dominant bacterial phyla in the feeding scars were Proteobacteria (51.00% ± 10.47%), Cyanobacteria (26.16% ± 12.57%), Bacteroidetes (14.70% ± 5.56%), and Planctomycetes (2.81% ± 1.13%) (Figure 2, Table S3).

**Figure 4:**
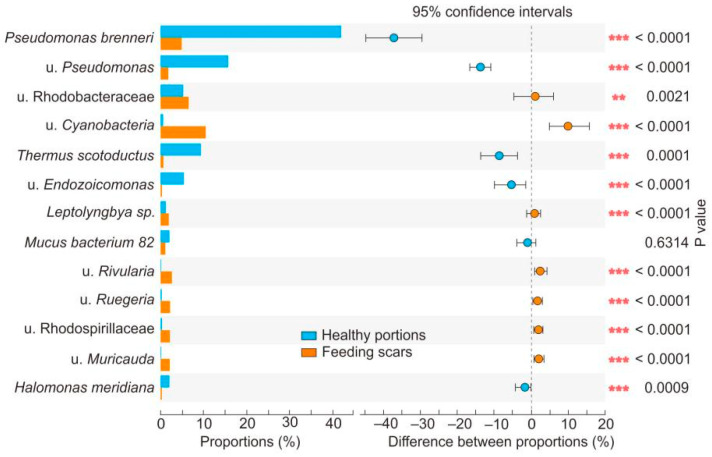
Student’s *t*-test results showing differences in bacterial abundance at the species level in *Pocillopora*, sampled from healthy portions and feeding scars. ** 0.001 < *p* < 0.01; *** *p* < 0.001.

At the bacterial genus level, the average relative abundances of *Pseudomonas*, *Thermus*, *Endozoicomonas*, and *Halomonas* in the healthy coral samples were significantly higher than those in samples of feeding scars (Figure 3, Figure 4, Table S4, Student’s *t*-test, *p* < 0.001). In contrast, the relative abundances of many genera, including Cyanobacteria, Rhodobacteraceae, Rhodospirillaceae, *Leptolyngbya*, *Muricauda*, *Vibrio*, *Ruegeria*, and *Rivularia*, in the feeding scars were significantly higher than in the healthy portions (Table S4, Student’s *t*-test, *p* < 0.001).

### 3.4. Beta-Diversity

**Figure 5:**
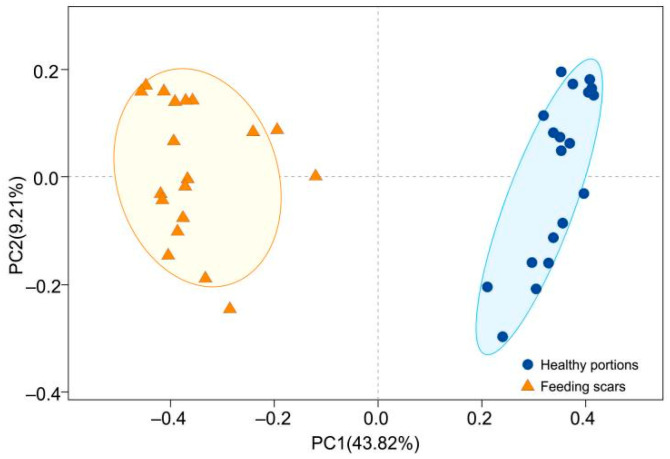
Principal coordinate analysis (PCoA) based on operational taxonomic units (OTUs) with grouping based on 60% similarity, as determined by complete linkage-cluster analysis. PC1 and PC2 explained 43.82% and 9.21% of the total variation, respectively.

PCoA, PERMANOVA, and ANOSIM were used to assess similarities between bacterial communities associated with the coral specimens at the I level (Table S5). PCoA analysis usiIOTU data based on Bray-Curtis metrics for healthy portions and feeding scars samples revealed that they were divided into two groups (Figure 5). Group I contained the samples collected from the healthy portions, and Group II contained the samples collected from the feeding scars. PERMANOVA (F = 28.37, R2 = 0.44, *p* = 0.001) and ANOSIM (*p* = 0.001) showed that the coral-associated microbial community compositions differed between the samples taken from the healthy portions and the feeding scars.

### 3.5. Microbial Gene Function Predictions

**Figure 6:**
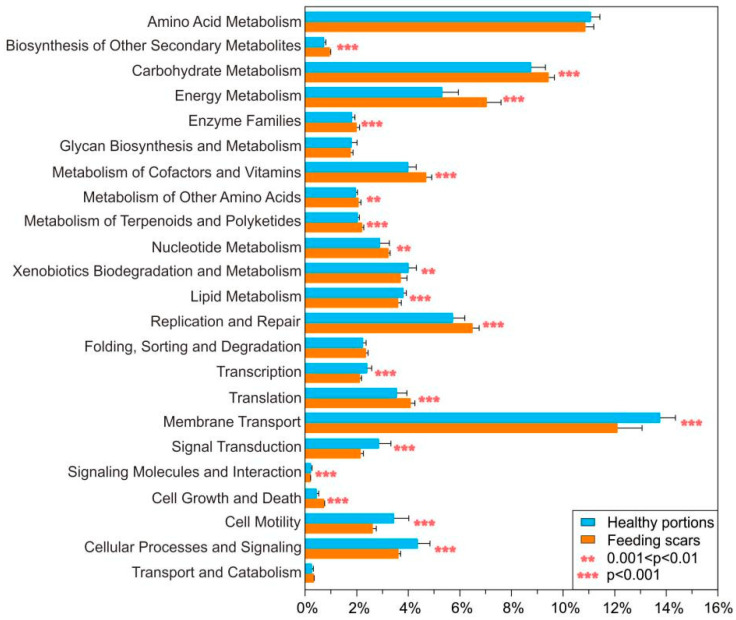
Mean relative abundance of each predicted functional trait in the KEGG pathways (level 2) using PICRUSt (V1.0.0) to analyze the predicted metagenomes, based on the 16S rRNA gene-sequencing data of coral-associated bacteria in *Pocillopora* sampled from healthy portions and feeding scars. The error bars indicate the standard deviation. ** 0.001 < *p* < 0.01; *** *p* < 0.001.

PICRUSt was used to predicted functions of bacterial genes of coral-associated bacteria, based on 16S rRNA sequencing data. A total of 21 predicted functional bacterial genic categories were analyzed from KEGG level 2 pathways (Figure 6, Table S6). Most bacterial genic categories showed significantly different average relative abundances of genes related to the KEGG terms, including amino acid metabolism, biosynthesis of other secondary metabolites, carbohydrate metabolism, energy metabolism, glycan biosynthesis and metabolism, metabolism of cofactors and vitamins, metabolism of other amino acids, metabolism of terpenoids and polyketides, nucleotide metabolism, xenobiotics biodegradation and metabolism, lipid metabolism, replication and repair, transcription, translation, membrane transport, signal transduction, cell growth and death, cell motility, and folding, sorting, and degradation (Figure 6, Table S6). Among these functional bacterial genic categories, the average relative abundances of genes related to amino acid metabolism, xenobiotic biodegradation and metabolism, lipid metabolism, membrane transport, signal transduction, and cell motility were significantly lower in samples obtained from healthy portions than those from feeding scars. In contrast, healthy coral samples showed significantly higher relative abundances of genes related to biosynthesis of other secondary metabolites, carbohydrate metabolism, energy metabolism, glycan biosynthesis and metabolism, metabolism of cofactors and vitamins, metabolism of other amino acids, metabolism of terpenoids and polyketides, nucleotide metabolism, replication and repair, folding, sorting and degradation, transcription, translation, cell growth, and death, when compared to that of coral scars (Figure 6, Table S6).

## 4. Discussion

COTS ingestion can dramatically affect the coral host and associated microbes [10,50]. COTS feeding can dramatically destroy coral holobionts within 3–5 h, resulting in the failure to recover and the death of numerous corals. Similar patterns of destruction have been observed with other coral predator animals, such as sea urchins, parrotfish, and members of the *Drupella* genus [6,8,51]. For example, Bessey et al. [8] investigated the ingestion of *Drupella cornus* on *Acropora spicifera* in Ningaloo Marine Park (Western Australia) and found that the aggregation feeding behavior of *Drupella cornus* directly led to the ingestion of entire coral tissues, resulting in large Symbiodiniaceae losses. Our study showed that *Pocillopora* feeding by COTS caused corals to lose most of their Symbiodiniaceae due to massive tissue loss.

In this study, COTS ingested *Pocillopora* corals and caused a large loss of coral tissue, thereby changing the microbial diversity and community. Results of the bacterial community structure showed that they had changed significantly no matter the level of bacteria phyla or genus. The relative abundance of *Pseudomonas*, *Thermus*, *Endozoicomonas*, and *Halomonas* bacteria in healthy portions was significantly higher than that in feeding scars. These bacteria usually live in the coral mucus layer and endoderm layer [30,52,53]. In contrast, Cyanobacteria, Rhodobacteraceae, *Leptolyngbys*, Rhodospirillaceae, *Muricauda*, *Ruegeria*, and *Vibrio* bacteria usually live in the coral mucus, endodermal layer, and skeletal surface [53,54], and the relative abundance of these bacteria in feeding scars is significantly higher than that in healthy portions. Drastic changes in the coral-associated bacterial community may affect the physiological function of the coral holobiont [22]. Many coral-associated bacteria, such as *Endozoicomonas* and Roseobacteriales, are considered beneficial to the coral host [25,26]. The potential role of these beneficial bacteria in biology are to transfer, spread, and regulate energy and promote protein supply and carbohydrate circulation to coral hosts [30,55]. Roseobacteriales involved in the sulfur cycle are generally considered to be obligate partners in symbiotic Symbiodiniaceae, which can increase the growth rate of Symbiodiniaceae [22,29]. In our study, COTS ingestion resulted in considerable tissue loss from *Pocillopora* corals, with a significant decrease in associated bacteria, including *Pseudomonas*, *Thermus*, *Endozoicomonas*, and *Halomonas*. In turn, some low-abundance bacteria were increased, which eventually resulted in significant changes in the coral-associated bacterial community.

Based on other corallivory, it is suggested that the ingestion of many corallivory has a great effect on a coral’s microbial community composition and diversity [50,51,56]. For example, Maher et al. [50] found that the feeding scars alone greatly affected changing microbial community composition and diversity of *Pocillopora meandrina* fed by corallivory of parrotfishes and puffer fishes. The relative abundance of Desulfovibrionaceae with feeding scars causes the significant change in this microbe from control corals with 0.09% ± 0.09% to 9.92% ± 6.30% (*p* < 0.01), while scarred treatments produced a high mean community diversity (0.67 ± 0.11), six times greater than that of the controls [50]. Moreover, Nicolet et al. [56] found that corallivorous invertebrate *Drupella* spp. create deep feeding scars that change microbial community composition and diversity of *Acropora muricata* and increase pathogens transmission. They found that *Drupella* spp. transmitted brown band disease to healthy corals in 40% of cases immediately following feeding on infected corals, and even in 12% of cases 12 and 24 h following feeding [56]. In our study results, it is similar to *Drupella* spp. that the bacterial community diversity of scars fed by COTS were higher than healthy portions, and feeding by COTS indeed greatly changed the composition and diversity of coral bacterial holobionts.

Microbial functional genic predictions suggested that the health state differed between feeding scars and healthy portions in *Pocillopora* corals. Using PICRUSt, which is highly effective in the functional predictions of metabolic pathways, genetic information processing, environmental information processing, and cellular processes of microbial communities [48], seems to be essential for the analysis of differences in functional predictions related to digestion in coral-associated bacterial communities between feeding scars and healthy portions in *Pocillopora* corals. The coral microbiome is enriched in several protein functions that support carbohydrate metabolism in coral holobionts, which reflect different environmental conditions of corals [27]. Based on the changes in the microbiome metabolic levels, significant differences were found in the functional characteristics of the coral-bacterial communities between feeding scars and healthy portions in *Pocillopora* corals due to a loss of the tissue, including amino acid metabolism, exogenous biodegradation and metabolism, lipid metabolism, membrane transport, signaling conduction, and cell motility.

In this study, numerous feeding scars of corals and significant changes in bacterial communities were observed after ingestion by COTS, which may affect the health state and survival of *Pocillopora* corals. For example, the COTS outbreak in Guam from 1967–1969 lasted for 30 months, resulting in the death of ~90% of all corals in the shallow water area of the 38 km coastline [13]. A COTS outbreak in the French Polynesia Islands and directional diffusion towards Moorea Island caused a 96% loss in the coral population [12]. After a COTS outbreak, numerous feeding scars provide areas for the growth of filamentous algae and pathogens such as *Vibrio coralliilyticus*, which may cause coral white plague disease [12,35]. Our research suggests that after *Pocillopora* corals were ingested by COTS, the relative abundances of several bacterial pathogens significantly increased in the feeding scars, which may have caused the healthy portions of the corals to become vulnerable to invasion by filamentous algae.

## Data Availability

The data used in this paper can be obtained from Electronic Supplementary Materials (ESM), and also can be found in the Dryad Dataset, https://doi.org/10.5061/dryad.37pvmcvhr, (accessed on 1 December 2021). The raw reads were deposited into the NCBI Sequence Read Archive database (Accession Number: PRJNA661173).

## Supplementary Materials

The following supporting information can be downloaded at: https://www.mdpi.com/article/10.3390/microorganisms10020207/s1, Table S1: Symbiodiniaceae density (×10^6^ cells cm^−2^) symbiotic with *Pocillopora* corals in the central SCS. Table S2: Sample information including number of sequences, number of coral-associated bacteria in different taxonomic. Table S3: The relative abundance of coral-associated bacterial community of coral samples on phylum level. Table S4: The relative abundance of coral-associated bacterial community of coral samples on genus level. Table S5: Similarity Percentage Analysis (SIMPER) for 25 most significant OTUs between the healthy portions and feeding. Table S6: Relative abundance of each predicted functional trait given in KEGG pathways (level 2).
